# Hipertensão Arterial como Mais uma Faceta do Tabagismo Passivo

**DOI:** 10.36660/abc.20250268

**Published:** 2025-06-04

**Authors:** Gilson Soares Feitosa

**Affiliations:** 1 Escola Bahiana de Medicina Salvador BA Brasil Escola Bahiana de Medicina, Salvador, BA – Brasil; 2 Hospital Santa Izabel da Santa Casa da Bahia Salvador BA Brasil Hospital Santa Izabel da Santa Casa da Bahia, Salvador, BA – Brasil

**Keywords:** Tabagismo, Tabagismo Passivo, Hipertensão Arterial

O tabagismo e a hipertensão arterial são fatores de risco reconhecidos para o desenvolvimento de doença cardiovascular. Por isso, esforços têm sido feitos no esclarecimento sobre seus potenciais malefícios bem como sobre os benefícios resultantes do seu controle junto à população em geral.

O tabagismo passivo tem recebido menos atenção quanto ao seu papel nocivo. Inicialmente, a atenção voltava-se para a irritação de rino e nasofaringe e, somente mais recentemente, a problemas relacionados ao pulmão^1^ e a doenças cardiovasculares.^2^

A proibição do tabagismo em locais públicos corroborou para a redução tanto do próprio tabagismo passivo como do tabagismo ativo. O Brasil se posiciona como um dos países mais bem-sucedidos nessa campanha. A frequência estimada de tabagismo no país varia entre 4% e 14,5%, com média de 9,1% para fumantes ativos, e 6,9% para fumantes passivos em ambiente domiciliar e 5,4% em ambiente de trabalho (segundo VIGITEL, 2021), apesar de leve aumento em 2023.^3^ No entanto, essas taxas ainda permanecem indesejavelmente elevadas.

Processos inflamatórios persistem por algum tempo após cessação do tabagismo, e a arteriosclerose parece ser permanente, mesmo após a sua interrupção.^4^ Dada a prevalência da hipertensão arterial estimada em 26,3%, sua interação com tabagismo assume grande relevância como fator potencializador de tais lesões. Embora se saiba que o tabagismo ativo se correlaciona com maiores taxas de hipertensão arterial, apesar de estudos recentes,^2,5^ tais informações não existem ou são escassas em relação ao tabagismo passivo.

No estudo publicado nos Arquivos Brasileiros de Cardiologia os autores utilizaram um grande banco de dados colhido por entrevista telefônica (VIGITEL), com amostras de todas as capitais brasileiras e no Distrito Federal. As amostras foram ajustadas quanto a fatores de confusão como sexo, idade, cor da pele, escolaridade, estado civil e região. Os resultados apontaram para uma associação entre tabagismo passivo e presença de hipertensão arterial numa magnitude semelhante à do tabagismo ativo de mais de um maço de cigarro ao dia.^6^

Em ex-tabagistas que permanecem com o tabagismo passivo, ou mesmo sem esse, há uma associação ainda mais forte com a hipertensão arterial. Uma curiosidade é a menor associação de hipertensão arterial com o tabagismo leve, de menos que um maço de cigarro por dia, sugerindo um efeito até protetor, talvez até como um amenizador do estresse.^7^ A literatura mostra achados conflitantes sobre esta relação de tabagismo leve com hipertensão arterial.

Após a cessação do tabagismo, o aumento de peso concomitante ao aumento da pressão arterial pode explicar uma aparente proteção do hábito de fumar,^8^ ou em alguns casos, mascarando-se uma hipertensão arterial. Contudo, no tabagismo ativo, mesmo que leve, há a inalação de ar circundante onde se encontram os elementos que determinam os males do tabagismo passivo. Os autores desse trabalho devem ser parabenizados pela construção de um conhecimento como o da significativa associação do tabagismo passivo com hipertensão arterial.

No entanto, a natureza da coleta de dados poderia em princípio apresentar alguns senões. Os dados foram obtidos a partir de informação voluntária dada pelos participantes por telefone, sem a possibilidade de verificação ou de medida objetiva da exposição. Não foram avaliados, por exemplo, marcadores como cotinina e carbohemoglobina, e outros, no sangue, na urina ou na saliva. A grande amostra populacional ameniza até certo ponto essa questão.

Assim, o estudo reforça uma importante associação do tabagismo passivo com a hipertensão arterial. Esse dado é particularmente importante para o momento em que o mundo se vê cercado de propaganda enganosa que procura estimular outras formas de tabagismo com provável igual malefício ao do uso do cigarro.^9^
