# Supplementary material for: Hipertensão Arterial como Mais uma Faceta do Tabagismo Passivo
Source: Arq Bras Cardiol. 2025 Jun 4;122(6):e20250268. [Article in Portuguese] doi: 10.36660/abc.20250268 (PMC12212392; doi:10.36660/abc.20250268)
Supplement: Translation

# Translation: Hypertension as another Facet of Passive Smoking

**DOI:** 10.36660/abc.20250268i

**Authors:** Gilson Soares Feitosa

**Affiliations:** 1 Brazil Escola Bahiana de Medicina, Salvador, BA – Brazil; 2 Brazil Hospital Santa Izabel da Santa Casa da Bahia, Salvador, BA – Brazil

**Keywords:** Smoking, Passive Smoking, Arterial Hypertension

Smoking and hypertension are recognized risk factors for the development of cardiovascular disease. Efforts have been made to clarify their potential harm, as well as the benefits resulting from their control among the general population.

Passive smoking has received less attention regarding its harmful role. Initially, the focus was on irritation of the rhino and nasopharynx, and only more recently, on lung-related problems^1^ and cardiovascular diseases.^2^

The ban on smoking in public places has contributed to the reduction of both passive smoking and active smoking. Brazil positions itself as one of the most successful countries in this campaign. The estimated frequency of smoking in the country ranges between 4% and 14.5%, with an average of 9.1% for active smokers and 6.9% for passive smokers in the home environment and 5.4% in the work environment (according to VIGITEL, 2021), despite a slight increase in 2023.^3^ However, these rates remain undesirably high.

Inflammatory processes persist for some time after smoking cessation, and arteriosclerosis appears to be permanent even after its interruption.^4^ Given the prevalence of hypertension estimated at 26.3%, its interaction with smoking assumes great relevance as a potentiating factor for such lesions. Although it is known that active smoking correlates with higher rates of hypertension, despite recent studies,^2,5^ such information does not exist or is scarce in relation to passive smoking.”

In the study published in the *Arquivos Brasileiros de Cardiologia*, the authors used a large database collected through telephone interviews (VIGITEL), with samples from all Brazilian capitals and the Federal District. The samples were adjusted for confounding factors such as sex, age, skin color, education, marital status, and region. The results pointed to an association between passive smoking and the presence of hypertension in a magnitude similar to that of active smoking of more than one pack of cigarettes per day.^6^

In former smokers who continue to smoke passively, or even without it, there is an even stronger association with arterial hypertension. One interesting fact is the lower association of arterial hypertension with light smoking, of less than a pack of cigarettes per day, suggesting a protective effect, perhaps even as a stress reliever.^7^ The literature shows conflicting findings about this relationship between light smoking and arterial hypertension.

After smoking cessation, the concomitant weight gain with increased blood pressure may explain an apparent protection of the smoking habit,^8^ or in some cases, masking hypertension. However, in active smoking, even if light, there is inhalation of surrounding air where the elements that determine the harms of passive smoking are found. The authors of this work should be congratulated for building knowledge such as the significant association of passive smoking with hypertension

However, the nature of data collection could, in principle, present some caveats. The data were obtained from voluntary information given by participants over the phone, without the possibility of verification or objective measurement of exposure. For example, markers such as cotinine and carboxyhemoglobin, among others, in blood, urine, or saliva were not evaluated. The large sample, however, mitigates this issue to some extent.

Thus, the study reinforces an important association between passive smoking and hypertension. This data is particularly important at a time when the world is surrounded by misleading advertising that seeks to promote other forms of smoking with likely equal harm to cigarette use.^9^
